# Manic Episodes in a Patient With Neurosyphilis Following Brief Discontinuation of Bipolar Medication

**DOI:** 10.7759/cureus.43604

**Published:** 2023-08-16

**Authors:** Hafiz Olatunde, Odiaka M Anombem, Aditya Mantha, Adeolu O Oladunjoye, Tyler Hudish

**Affiliations:** 1 Psychiatry and Behavioral Sciences, Meharry Medical College, Nashville, USA; 2 Research, Methodist Health System, Dallas, USA; 3 Psychiatry and Behavioral Sciences, The University of Colorado School of Medicine, Aurora, USA; 4 Psychiatry, Baylor College of Medicine, Houston, USA; 5 Medical Critical Care, Boston Children's Hospital, Boston, USA; 6 Psychiatry, Rocky Mountain Regional Veterans Affairs Medical Center, Aurora, USA

**Keywords:** lithium toxicity, lithium, mania, neurosyphilis, bipolar 1

## Abstract

Bipolar disorder is a mood disorder resulting in episodes of either mania or hypomania. The episodes can manifest themselves as a period of abnormally and persistently elevated mood, abnormally and persistently increased activity or energy, distractibility, insomnia, grandiosity, flight of ideas, increased activity, pressured speech, and racing thoughts. Neurosyphilis is a progression of syphilis infection involving the brain, meninges, or spinal cord. The interaction between bipolar disorder and neurosyphilis has not been extensively studied, but it has been theorized that neurosyphilis can exacerbate mood disorders. This case study details a patient with concurrent late-onset bipolar disorder and neurosyphilis and how the discontinuation of bipolar medication resulted in an acute manic episode. In addition, this case underscores the importance of differentiating the presenting symptoms between bipolar disorder and neurosyphilis.

## Introduction

Bipolar disorder is characterized as a mood disorder resulting in periods of manic or hypomanic states [1]. Symptoms of mania include a period of abnormally and persistently elevated mood, abnormally and persistently increased activity or energy, distractibility, insomnia, grandiosity, flight of ideas, increased activity, pressured speech, and racing thoughts [2]. The DSM-5-TR (Diagnostic and Statistical Manual of Mental Disorders, Fifth Edition, Text Revision) lists different subtypes of bipolar disorder categorized as Bipolar I if the criteria for manic episodes are met and Bipolar II if the criteria for hypomania are met [1]. Treatment for bipolar disorder includes a multifocal approach involving psychotherapy, such as cognitive behavioral therapy, and pharmacotherapy, such as lithium, valproic acid, and atypical antipsychotics [2]. 

Neurosyphilis is a disease involving the brain, meninges, or spinal cord and could occur at any time of the infection from months to years [3]. Neurosyphilis is categorized as asymptomatic, meningeal, meningovascular, general paresis, and tabes dorsalis neurosyphilis [3]. General paresis and tabes dorsalis can occur a few years after an untreated syphilis infection and result in personality and mood disturbances as well as loss of reflexes and impaired gait and balance [3]. General paresis can also present with labile mood, irritability, and psychosis. Prognosis depends mainly on the length of time the neurosyphilis has been untreated, and though an individual can improve with ceftriaxone treatment, they may not return to the initial baseline [4]. 

The interaction of bipolar disorder and neurosyphilis is not entirely understood [5]. There have been previous cases demonstrating neurosyphilis presenting as manic episodes with psychosis [6,7]. This case study describes a 69-year-old with a long-standing neurosyphilis disorder, a recent bipolar diagnosis, and an acute decompensation after briefly discontinuing lithium and valproic acid maintenance therapy. This case raises the question of the late onset of bipolar disorder, the presence of both bipolar disorder and neurosyphilis, and finally, the therapeutic approach to acute decompensated mania.

## Case presentation

A 69-year-old man presented in the emergency department with complaints of fever, diarrhea, shortness of breath, and tremors in both hands. The tremors in both hands started 10 years ago when he was diagnosed with neurosyphilis. Propranolol was previously used to treat the tremors but was discontinued after an episode of bradycardia. He has a past history of bipolar disease with psychotic features, which was currently being treated with lithium and valproic acid. He also had a past history of prostate malignancy. On examination, he was febrile with a temperature of 101.5°F, pulse rate of 98 beats per minute, respiratory rate of 19 breaths per minute and blood pressure was 109/71 mmHg. His chest was clear on auscultation and he exhibited tremors in his upper extremities with cogwheel rigidity. Laboratory tests showed leukocytosis of 14.5 × 10^9^/L (reference range 4.41-10.05 × 10^9^/L], elevated CRP of >300 mg/L (reference range <5 mg/L), and elevated erythrocyte sedimentation rate (ESR) of 82 mm/hr (reference range 0-20 mm/hr). A diagnosis of E. coli bacteremia secondary to a prostatic abscess was made and he was admitted to the medical unit. An incision and drainage of the prostatic abscess were performed, and he was also started on intravenous ceftriaxone and continued his lithium at 1200 mg and valproic acid at 2500 mg.

On the first day of admission, the fever, diarrhea, and shortness of breath had subsided, but the tremors were still present. On examination, his temperature was 98.3°F, pulse rate was 76 beats per minute, respiratory rate was 16 breaths per minute, and blood pressure of 122/80 mmHg. The tremors in the upper extremities had worsened, and this raised concerns about lithium toxicity. The serum lithium level was elevated at 2.1 mmol/L (reference range 0.6-1.2 mmol/L), serum valproic acid was 71 mcg/mL (reference range 50-100 mcg/mL), and serum creatinine level was also elevated at 2.11 mg/dL (reference range 0.67-1.17 mg/dL). Due to the worsening of the tremors, supratherapeutic serum lithium levels, and acute kidney injury, the lithium dose was withheld on the first day of admission, and the patient was continued on valproic acid.

On the second day of admission, oral lithium was restarted at half the previous dose of 600 mg. On the fifth day of admission, due to the persistent worsening of the tremors and supratherapeutic serum lithium levels, lithium was discontinued, and valproic acid was continued at 2500 mg. On the tenth day of admission, valproic acid was discontinued due to hyperammonemia and elevated liver enzymes, and he was started on olanzapine at a starting dose of 2.5 mg and was titrated to 5 mg daily due to the renal effects of lithium.

On the eleventh day of admission, the patient complained of some manic symptoms. The manic symptoms did not emerge until the sixth day following the discontinuation of lithium and one day after discontinuing valproic acid. He complained of having racing thoughts which started earlier that morning, and on examination, his mood was labile. By the evening of the eleventh day, the manic symptoms had worsened and included pressured speech, insomnia, and tangential thoughts. Valproic acid was restarted at a dose of 1700 mg at night and titrated up to 2500 mg over three days. Olanzapine dosage was then increased over this three-day timeframe to 30 mg total per day divided four times throughout the day. The manic symptoms persisted for four more days, and lithium was eventually restarted at 450 mg twice a day. Manic symptoms continued for an additional four days after the lithium restart before resolution eventually began. His insomnia improved, and his thought content became less tangential. He was continued on the above doses of lithium, valproic acid, and olanzapine and was then admitted to the psychiatric service for further treatment of manic symptoms. Insight and judgment improved from poor to fair, and pressured speech gradually resolved. Olanzapine was then tapered off to rule out antipsychotic medication contributing to tremor. He did not endorse any change in mood or relapse into mania upon discontinuation of olanzapine. The tremor resolved to baseline levels, and serum lithium was within the therapeutic range at 0.9 mmol/L. The patient was discharged 61 days after presentation to the emergency department. He was discharged on 900 mg of lithium daily and 2500 mg of valproic acid daily. 

At the two-month follow-up, the lithium dose was decreased to 750 mg daily due to serum lithium levels of 1.6 mmol/L (reference range 0.6-1.2 mmol/L). Repeat serum lithium levels at this dose were 1.0 mmol/L (reference range 0.6-1.2 mmol/L) at subsequent follow-up. Valproic acid was decreased to 2000 mg at a three-month follow-up in an effort to reduce upper extremity tremors. Manic symptoms were not reported at the subsequent four-month follow-up.

## Discussion

Neurosyphilis and bipolar disorder are two distinct medical conditions that both affect the brain and can cause significant impairment in daily functioning. While they are different in many ways, there are some similarities between the two conditions. One similarity between neurosyphilis and bipolar disorder is that they both can produce mood symptoms [8]. Periods of mania, depression, and psychosis can be symptoms of both diseases [8].

While there are some similarities between the two conditions, important differences set them apart. One of the key differences between neurosyphilis and bipolar disorder is their underlying causes. Neurosyphilis is caused by the bacterium *Treponema pallidum* [9], while bipolar disorder, on the other hand, is thought to be caused by a combination of genetic, environmental, and neurochemical factors [10]. Another difference between the two conditions is their clinical presentation. Neurosyphilis can cause various neurological symptoms, such as headaches, difficulty with coordination, and vision changes [11]. In contrast, bipolar disorder primarily manifests as a mood disorder characterized by episodes of mania and depression, as well as changes in behavior and energy levels [12]. The treatment approaches for neurosyphilis and bipolar disorder also differ significantly. Neurosyphilis is typically treated with antibiotics to eliminate the bacterium and prevent further damage to the nervous system [13]. In contrast, bipolar disorder is treated with a combination of medications, therapy, and lifestyle changes to manage symptoms and improve overall functioning [10].

The link between neurosyphilis and bipolar disorder is not fully understood, but evidence suggests that neurosyphilis can cause or exacerbate mood disorders, including bipolar disorder [6]. One theory suggests that *Treponema pallidum* has the potential to infiltrate the brain directly, initiating a cascade of events that ultimately leads to the release of specific biomarkers which disrupts the normal functioning of neurotransmitters at nerve junctions within the brain which can cause clinical features of bipolar disorder [14]. 

This patient with a history of neurosyphilis experienced symptoms of mania on the eleventh day of admission. These symptoms were not likely due to neurosyphilis as he had received a full course of antibiotic treatment at the time of his neurosyphilis diagnosis many years prior. Also, his current diagnosis is not likely due to neurosyphilis because of the absence of other symptoms and signs of the disease, such as the absence of ataxia, positive Romberg sign, cranial nerve palsies, or paresthesia. He also received a long course of intravenous ceftriaxone for four weeks during this admission for the abscess in his prostate. This course of antibiotics would have simultaneously treated the neurosyphilis if the disease had been present. 

About 54% of adults with severe mental health disorders experience withdrawal reactions after abruptly discontinuing treatment or gradually tapering off psychiatric medications [15]. As seen in our patient, abrupt discontinuation of both lithium and valproic acid can result in a relapse of bipolar disorder [16]. Generally, the dose of lithium and valproic acid should be gradually tapered off over several weeks to minimize the risk of withdrawal symptoms or relapse [16,17]. Due to this patient's acute kidney injury, hyperammonemia, and elevated liver enzymes, both lithium and valproic acid were discontinued over a very short period, likely contributing to the relapse of his bipolar symptoms. In cases such as this one, where abrupt discontinuation of bipolar medication is unavoidable, patients should be closely monitored for symptoms of bipolar disorder. Other bipolar medications, such as antipsychotic medications, may be necessary, and delays in such substitutions should be minimized.

## Conclusions

In conclusion, while neurosyphilis and bipolar disorder share some similarities, they are fundamentally different conditions with separate causes and treatments. Making an accurate diagnosis and providing the appropriate treatment for each condition is vital to achieving the best possible outcomes. Also, when there is an indication to discontinue bipolar medication, it should be done gradually, and after discontinuation of bipolar medication, patients should be closely monitored for symptoms and signs of bipolar disorder.
